# Injectable platelet-mimicking silk protein-peptide conjugate microspheres for hemostasis modulation and targeted treatment of internal bleeding

**DOI:** 10.1186/s12951-025-03180-w

**Published:** 2025-02-20

**Authors:** Yajun Shuai, Yu Qian, Meidan Zheng, Chi Yan, Jue Wang, Peng Wang, Jie Wang, Chuanbin Mao, Mingying Yang

**Affiliations:** 1 Institute of Applied Bioresource Research, College of Animal Sciences, Zhejiang University, Hangzhou, 310058 China; 2 Key Laboratory of Silkworm and Bee Resource Utilization and Innovation of Zhejiang Province, Zhejiang University, Hangzhou, 310058 China; 3 Department of Biomedical Engineering, The Chinese University of Hong Kong, Sha Tin, Hong Kong SAR China

**Keywords:** Hemostatic agents, Wound closure, Micro-nanoparticles, Injectable microsphere, Targeted therapy

## Abstract

Uncontrolled deep bleeding, commonly encountered in surgical procedures, combat injuries, and trauma, poses a significant threat to patient survival and recovery. The development of effective hemostatic agents capable of precisely targeting trauma sites in deep tissues and rapidly halt bleeding remains a considerable challenge. Drawing inspiration from the natural hemostatic cascade, we present platelet-like microspheres composed of silk fibroin (SF) and thrombus-targeting peptides, engineered to mimic natural platelets for rapid hemostasis in vivo. These peptide/SF hemostatic microspheres, formulated using a freezing self-assembly technology, closely resemble natural platelets in terms of size, shape, and zeta potential. Moreover, they exhibit favorable cytocompatibility, hemocompatibility, and anti-cell adhesion. Assessment of fibrin polymerization revealed that these hemostatic microspheres possessed enzymatic physiological functions, similar to activated platelets, facilitating platelet adhesion, fibrin binding, and wound-triggered hemostasis. Notably, these hemostatic microspheres rapidly target the bleeding site in vivo within 5 min, with minimal dispersion elsewhere, persisting after blood clot formation. Furthermore, these microspheres exhibit favorable metabolic kinetics, with 71% degradation occurring within one-day post-subcutaneous injection. Histological assessment revealed well-preserved organ structures and minimal inflammatory responses at 14 d post-injection, supporting their long-term biocompatibility. Importantly, they can be injected and targeted into damaged blood vessels, selectively binding to fibrin and forming blood clots within 2 min, resulting in a 74% reduction in bleeding volume compared to SF microspheres alone. Therefore, these injectable SF-based hemostatic microspheres emerge as promising candidates for future rapid hemostasis in tissue injuries.

**Graphical Abstract:**

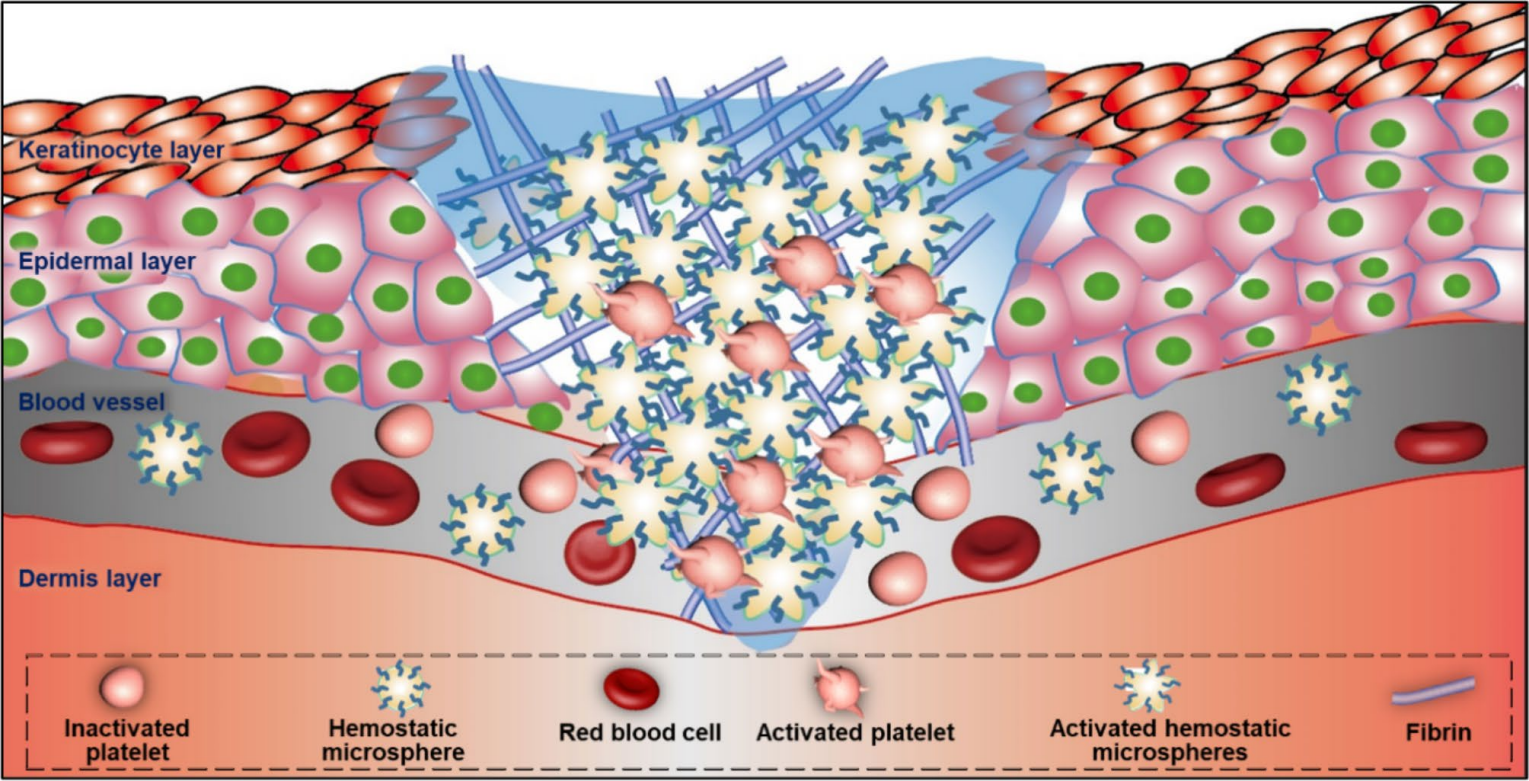

**Supplementary Information:**

The online version contains supplementary material available at 10.1186/s12951-025-03180-w.

## Introduction

Hemostatic agents have garnered considerable attention in the fields of trauma emergencies, surgery, and military medicine due to their critical role in controlling bleeding and saving lives [1, 2]. Recently, various polymer-based hemostatic agents, including fibers, membranes, aerogels, sponges, and hydrogels, have been extensively investigated [3–7]. These materials are engineered to interact with blood components, promoting clot formation and providing a mechanical barrier to bleeding in moist environments. While these hemostatic materials showcase excellent performance in managing surface wounds, they frequently suffer from inherent limitations, such as suboptimal adhesion to trauma sites, non-targeted thrombus formation, an increased risk of infection, and concerns regarding biosafety. Particularly, traditional hemostatic materials encounter challenges in safely achieving rapid hemostasis at bleeding sites, especially within complex internal and deep tissues in vivo [8, 9]. Therefore, there exists an imperative need for the development of intravenous hemostatic materials characterized by robust biocompatibility, effective targeting of hemorrhaging sites, and the capacity for prompt hemostasis to address these limitations.

The process of blood coagulation in vivo is dynamic, transitioning from a liquid state to a non-flowing aggregation state primarily through activated platelet deformation, mediated by protein-protein interactions [3, 4]. Platelets, pivotal in primary hemostasis, have been harnessed as hemostatic agents to facilitate rapid wound closure [10, 11]. However, existing platelet-based hemostatic agents encounter notable challenges in clinical practice, including susceptibility to contamination, stringent storage requisites, restricted hemostatic efficacy, and a limited shelf-life (5 to 7 d) [12, 13]. Although preservation techniques, such as freeze-drying, can extend the shelf-life of platelets [14], limitations related to platelet sourcing and the risk of immunogenicity hinder their widespread utilization [15, 16]. Recently, innovative strategies to develop platelet-imitating agents have emerged, focusing on replicating the morphology and specific physiological functions of platelets [17–20]. This strategy involves creating micro- or nanoparticles from materials such as liposomes [20] or polymers [19, 21], which are designed to closely resemble natural platelets and their hemostatic functions following vascular injury [22]. Compared to natural platelets, these synthetic materials offer advantages such as larger-scale production and extended shelf lives, demonstrating significant clinical potential [23]. Despite promising advancements, the hemostatic modulation process of these microspheres in vivo and in vitro remains poorly understood. Moreover, current methods often rely on chemical grafting of hemostatic factors onto platelet substitutes, potentially leading to concerns regarding biological toxicity, hemostatic peptide leakage, and non-targeted coagulation in vivo.

*Bombyx mori* silk fibroin (SF) is a natural polymer known for its excellent biocompatibility, plasticity, and biodegradability [24, 25]. Its versatility has gained attention across diverse fields such as regenerative medicine [26, 27], cancer therapy [28, 29], stem cell therapy [30], and gene therapy [31]. The molecular chain of SF consists of hexapeptide fragments (GAGAGS) and non-repeating sequences with distinct physical and chemical properties [32]. The hydrophobic crystalline regions predominantly maintain an organized β-sheet configuration, while the hydrophilic, non-crystalline regions, encompass amino acids with larger side chains. Responsive to factors, such as hydration, organic solvents, temperature, and pH, the molecular chain structure of SF undergoes stimuli-responsive spatiotemporal and conformational changes [29, 33]. Macroscopically, SF materials exhibit transformational behaviors, including self-deformation [34], self-assembly [35], and self-healing [33]. These properties position SF as a promising candidate for developing platelet substitutes that mimic the physiological function of platelets. However, to our knowledge, the utilization of SF particles for imitating platelet functions and achieving targeted hemostasis has not yet been explored.

Here, we employed a one-pot method, known as freezing self-assembly technology [36], to synthesize SFMPs and optimize their production as artificial platelets. To enhance the targeting and adhesion effect of the microspheres to injured vascular sites, three tissue-targeting peptides, namely RD (RGD), TI (TRYLRIHPQSQVHQI), and LD (LPCDYYGTCLD), were conjugated with SF. RD is a tripeptide derived from the extracellular matrix (ECM) that specifically binds to integrins, facilitating cell adhesion to the ECM. Additionally, RD functions as an antagonist to the platelet glycoprotein IIb/IIIa receptor [37], aiding in the modulation of platelet aggregation. The LD peptides have demonstrated specific binding to fibrin [38], while the TI peptide is a ligand that interacts with von Willebrand factor, facilitating binding to activated platelets and aggregated fibrin [39]. During the freezing self-assembly process, these peptide molecules were immobilized within the SF molecular chain as they transformed from the amorphous to the crystalline region [29]. As a result, the final product not only possesses the morphological characteristics of platelets but also demonstrates the ability to recognize and adhere to bleeding sites, thus effectively contributing to the process of artificial hemostasis. The design and fabrication of these microspheres provide a novel approach for the development of more effective hemostatic agents, particularly in the context of complex trauma, with significant clinical application potential.

## Materials and methods

### Self-assembly of SF microspheres

SF aqueous solution was extracted from previously established methods [32]. Briefly, silkworm cocoons sourced from Shandong Academy of Sericulture, China, were degummed twice using a Na_2_CO_3_ (Macklin, China) aqueous solution, followed by dissolution in LiBr (Sinopharm Chemical Reagent Co., Ltd, China) aqueous solution, and dialyzed for 80 h to obtain SF aqueous solution. To prepare SFMPs, the SF solution was diluted to a concentration of 2 wt%. Ethanol was then added to 8 mL of the SF solution at different volumes (1, 2, or 4 mL) and stirred for 10, 30, and 50 min to promote the self-assembly of SF. The resulting SF/ethanol solution was then stored at -20℃ for 24 h. After freezing, the SF/ethanol mixture was gradually thawed at room temperature, resulting in a milky white solution. This solution was centrifuged at 6000 r/min for 14 min to collect the precipitate, which was then dispersed and further centrifuged using a mixed alcohol-aqueous solution. Lastly, dry SFMP powder was obtained by freeze-drying.

### Preparation of hemostatic microspheres

Hemostatic microspheres were prepared using RD, TI, and LD peptides, each with a purity of 95% (GL Biochem Ltd., Shanghai). The preparation process of hemostatic microspheres was similar to that of SFMP. Prior to the addition of ethanol, 5 mg of each peptide (RD, TI, or LD) was mixed with 8 mL of the 2 wt% SF solution for 30 min. Following this, the SF/peptide solution was mixed with 2 mL of ethanol, pre-assembled for 5 min, and then frozen for 24 h at -20℃. After thawing, the solution was centrifuged at 6000 r/min for 14 min, discarding the supernatant. The resulting microspheres were washed with deionized water, ultrasonically dispersed, and subjected to a centrifugation-washing process twice to obtain the hemostatic microspheres. Hemostatic microspheres containing RD, TI, and LD peptides were denoted as RDMP, TIMP, and LDMP, respectively. To obtain dry hemostatic microsphere powders, the aqueous solution of microspheres was freeze-dried.

### Characterization of SFMP and hemostatic microspheres

Morphological analysis of the microsphere powders was performed using scanning electron microscopy (SEM, SU8010, Hitachi). To evaluate the capability of SFMPs for peptide loading, fluorescein isothiocyanate (FITC, 46425, Thermo Scientific) was used as an indicator. FITC-locked peptides were prepared by dissolving FITC in dimethylformamide (DMF) and adding it to a 2 wt% SF solution at a final concentration of 0.2 mg/mL. The mixture was stirred for 0.5 h at room temperature. Fourier-transform infrared spectroscopy (FTIR, SHIMADZU 8400s) was used to analyze the structure characteristics and crystallinity of SFMPs and hemostatic microspheres. FITC-SFMP, FITC-RDMP, FITC-TIMP, and FITC-LDMP were prepared as described in the SFMP synthesis method. The morphology of FITC-loaded hemostatic microspheres was visualized by confocal laser scanning microscopy (CLSM, ZEISS LSM780, German). For assessing the encapsulation efficiency of the TI peptide, Cyanine5 NHS Ester (Cy5, AAT Bioquest, Inc.)-labeled hemostatic microspheres were prepared following the same protocol used for FITC-labeled microspheres. A standard curve for Cy5 was constructed to calculate the Cy5 concentration by measuring the absorption peak at a wavelength of 649 nm. The Cy5-TIMP powder was dissolved in 1300 µL of water and centrifuged at 4000 r/min for 10 min. A 100 µL sample of the supernatant was measured for optical density (OD) at 649 nm. The encapsulation efficiency was calculated using the formula:

$$\:\text{E}\text{n}\text{t}\text{r}\text{a}\text{p}\text{m}\text{e}\text{n}\text{t}\:\text{e}\text{f}\text{f}\text{i}\text{c}\text{i}\text{e}\text{n}\text{c}\text{y}=\frac{1-Free\:Cy5\:concentration}{Total\:Cy5\:concentration}\times\:100\text{\%}$$

For zeta potential and particle size analysis, 1 mg/mL of aqueous hemostatic microspheres were incubated for 30 min, followed by testing for zeta potential and particle size distribution using a Zetasizer (NANO-ZS90, Malvern Instruments, UK).

### Biocompatibility test

The biocompatibility of the hemostatic microspheres was evaluated using mouse fibroblasts (L929 cells). A total of 100 µL of L929 cell suspension was seeded into a 96-well plate and cultured for 24 h at 37℃. After that, sterile microsphere powder was added to a fresh Dulbecco’s Modified Eagle Medium (DMEM, Thermo Fisher Scientific Inc.) to create a 5 mg/mL mixed solution. Then, 10 µL of this solution was added to the wells containing the cells, while the control group received 10 µL of phosphate-buffered saline (PBS) in place of the microsphere solution. Cell viability was assessed using the Cell Counting Kit-8 (CCK-8 assay, Beyotime Co. Ltd., China) by measuring absorbance at 450 nm with a microplate reader.

To directly observe the effects of hemostatic microspheres on cell adhesion and viability, the hemostatic microspheres were dispersed in a diluted SF solution to form a bilayer structure of the microsphere-based SF coating. Specifically, SFMP or hemostatic microspheres were incorporated into a 0.5 wt% SF solution. Then, 200 µL of this mixture was dropped on a glass slide and allowed to ventilate for 24 h, forming a microsphere-anchored film. Following ventilation, the film was immersed in an 80% (V/V) ethanol aqueous solution for immobilization and sterilization. The adhesion and viability of the cells on microsphere films were evaluated using the Live/Dead Cell Double Staining Kit (Yeasen Biotechnology Co., Ltd.) after 12 h and 3 d of culture. To simulate blood flow conditions, the non-fixed stained cells on microsphere-based SF coatings were subjected to horizontal shaking at a speed of 60 r/min to dislodge loosely adherent cells. Microscopic imaging (Eclipse Ti-E, Nikon, Japan) was performed to visualize the stained cells, with live cells appearing yellow-green (excitation at 490 nm) and dead cells appearing red (excitation at 545 nm). Adhered cell counts were quantified with ImageJ software.

### Hemolytic test of hemostatic microspheres

Anticoagulated blood was prepared using an anticoagulant citrate dextrose (ACD) solution containing sodium citrate, citric acid, and glucose, following previously reported protocols [40]. Diluted rat blood was prepared by adding 5 mL of the ACD anticoagulant. In each experiment group, 2 mL of PBS and 5 mg of microspheres were mixed and incubated for 24 h. For controls, 2 mL of PBS was used as a negative control, while 2 mL of deionized water served as the positive control. Then, 0.25 mL of diluted blood was added to each tube, mixed thoroughly, placed at 37℃ for 60 min, centrifuged at 3000 r/min for 5 min, and the supernatant was measured at 545 nm. The hemolytic ratio (HR) was calculated as follows:

$$\:\text{H}\text{R}\left(\text{\%}\right)=\frac{OD\left(Sample\right)-OD(-)}{OD\left(+\right)-OD(-)}\times\:100\text{\%}$$

Where OD (+) represents the OD of the positive group, OD ($\:-$) means the OD of the negative group, and OD (Sample) represents the OD of the microsphere groups.

### Platelet adhesion test

The platelet-rich plasma (PRP) was harvested from Sprague Dawley (SD, ♂, ≈ 250 g) rats for the platelet adhesion test on the surface of the double-layer microsphere film described previously. To prepare PRP, rat blood was centrifuged at 900 r/min for 12 min to isolate the supernatant. The PRP solution was diluted 10-fold with normal saline before use. A total of 0.5 mL of diluted PRP was added and kept at 37℃ for 2 h. After incubation, the microsphere films were rinsed with normal saline to remove unadhered platelets and impurities. The adhered platelets were then treated with glutaraldehyde solution for 1 h, followed by dehydration in gradient concentrations of ethanol. The adhesion morphology of platelets on the surfaces of microsphere films was observed using SEM (XL30-E, Philips), and platelet count was calculated by analyzing the obtained images.

### In vitro evaluation of fibrin polymerization and clot reaction

#### Fibrin polymerization assay

A mixture containing 2 mg/mL of fibrinogen labeled with Alexa Fluor 647 (Thermo Fisher Scientific Inc., U.S.A.), 10 mM CaCl_2_, and 0.2 IU/mL of thrombin (Merck KGaA, Darmstadt, Germany) was prepared in a tube. FITC-labeled microspheres (3 mg/mL) were added to the reaction mixture and incubated at 25–37°C. After 30 min of polymerization, fibrin formation and fiber structure were visualized under a CLSM (ZEISS LSM780, German). The binding kinetics between the hemostatic microspheres and fibrin were monitored by a microplate reader at an OD of 405 nm, and plotted using Origin software. The morphology of the dried microspheres/fibrin composite was observed by SEM (SU8010, Hitachi).

#### In vitro microsphere-mediated clotting assays

To investigate the impact of hemostatic microspheres on coagulation in vitro, platelet-poor plasma (PPP) was isolated from whole rabbit blood. PPP was prepared by centrifuging blood with anticoagulant at 1000 r/min for 10 min, followed by a second centrifugation at 3000 r/min for 20 min to collect the PPP. For the microsphere-mediated clot reaction, 50 µL of microspheres (2 mg/mL) was mixed with 20 µL of thrombin solution (20 IU/mL, Merck KGaA, Darmstadt), and 30 µL of PPP was added. The mixture was then incubated at 37℃ for 30 min. Kinetic changes in the clotting process were recorded using a microplate reader at OD 405 nm. Post-reaction, the fluorescence distribution of the FITC-SFMP and the FITC-RDMP groups was examined using CLSM (ZEISS LSM780, German). Additionally, the resulting PPP/microsphere-mediated clots were collected, freeze-dried, and observed for morphology using SEM. Effective porosity of blood clots was calculated by ImageJ software.

### Targeting, degradation and histocompatibility evaluation of hemostatic microspheres in vivo

#### Targeting at the wound site

For the targeting experiment, the microspheres were diluted in PBS at a concentration of 2 mg/mL. Balb/c mice (♀, ≈ 20 g) were utilized for the experiment. Mice were anesthetized using inhalation anesthesia (3.5% isoflurane mixed with oxygen) from an anesthesia machine (VT-110 small animal anesthesia machine). Once anesthetized, 80 µL of Cy5, Cy5-SFMP, Cy5-RDMP, Cy5-TIMP, and Cy5-LDMP were injected intramuscularly into the dorsal region, allowing for a circulation time of 5 min. Then, the right femoral artery was surgically exposed and incised to induce bleeding. At 5 min, 3, 12, and 36 h post-injection, whole-body fluorescence imaging of the mice was performed using a fluorescence imaging system (Caliper, IVIS Spectrum) to observe the distribution of microspheres within the mice.

#### Degradation of microsphere solutions and hydrogels in vivo

To investigate in vivo degradation, 80 µL of hemostatic Cy5- labeled MP solutions were subcutaneously injected into the backs of the mice. Imaging to track degradation was conducted at 0, 1, 3, 6, and 10 d post-injection. Meanwhile, to simulate the degradation of coagulation clots in vivo, the 2 mg/mL of microsphere solution was ultrasonic for 10 min, resulting in an injectable microsphere hydrogel formed through a suction and blowing process with a syringe. This hydrogel was then injected into the subcutaneous area to investigate biodegradation. Fluorescence signal intensity—quantified as the number of photons detected from the region of interest (ROI) in the subcutaneous dorsal area—was recorded via the fluorescence imaging system (Caliper, IVIS Spectrum), using units of radiation (Photons/s and photons/cm²/ steradian [sr]). Then the ROI areas were quantitatively analyzed using Aura software.

#### Metabolism and histocompatibility of microspheres in vivo

For tracing the metabolism of the microspheres in vivo, 80 µL of Cy5-labeled microsphere gels were injected subcutively into the dorsal region of the mice, with PBS serving as the negative control and Cy5 solution as the positive control. After 36 h, the mice were euthanized, and subcutaneous tissue at the injection site was collected for in situ fluorescence imaging (Cy5 appearing pink, DAPI appearing blue) to observe the retention and distribution of the microspheres in metabolizing organs (heart, liver, lungs, and kidneys).

Additional samples were collected from the heart, liver, lungs, and kidneys after 36 h and again at 14 d post-injection for H&E staining to assess histocompatibility of these metabolic organs. Concurrently, the subcutaneous tissue was subjected to immunofluorescence co-staining (CD68 in green, TNF-α in red, and DAPI in blue). The tissue samples were first incubated with CD68 primary antibody (1:200, Booster, BA3638) and TNF-α primary antibody (1:800, Bioss, bs-10802R), followed by incubation with TYR-488 (PN0100, Pinuofei Biological) and TYR-555 (PN0101, Pinuofei Biological), respectively. Nuclear staining was performed using DAPI (PN0015, Pinuofei Biological). Tissue sections were prepared and imaged using a fluorescence scanner (3DHISTECH, PANNORAMIC MIDI SP8) to assess tissue compatibility (Pinofei Biological). All animal experiments were approved by the Animal Welfare and Ethics Committee of Zhejiang University.

### Hemostasis effect of hemostatic microspheres in vivo

To evaluate the hemostatic efficiency in vivo, a rat femoral vein injury model was created. SD rats (♂, ≈ 250 g) were anesthetized using inhalation anesthesia (4% isoflurane/oxygen) from an anesthesia machine (VT-110 small animal anesthesia machine). Then, SD rats weighing approximately 300 g were anesthetized by inhalation anesthesia, shaved, disinfected, skin bluntly separated, and the femoral artery sheath was exposed. 700 µL of microsphere/PBS solution (2 mg/mL) was injected into the femoral vein and circulated in vivo for 5 min. The control group was administered an equivalent volume of PBS solution. Subsequently, the blood vessel was exposed to 5 mm in length. A 22-gauge needle was used to slide along the longitudinal axis of the blood vessel to create a 3 mm-long incision. Bleeding was monitored by absorbing the outflowing blood with a degreasing cotton ball every 30 s and weighing it. Finally, the rats were sacrificed, and the damaged vessel tissues were separated for tissue slice and Martius-scarlet-blue (MSB, G2040, Solarbio) staining for fibrin in adjacent vessel. All animal experiments were approved by Zhejiang University’s Animal Welfare and Ethics Committee, and the animals were treated per relevant regulations.

### Statistical analysis

Statistical analysis was performed with the GraphPad Prism 8.0 (GraphPad Software, USA). Results were collected in triplicate unless otherwise specified and expressed as mean ± standard deviation (Mean ± SD). Sample pairs were analyzed with the Student’s t-test, and one-way ANOVA analysis assessed statistical differences among groups. An asterisk signifies statistical significance as follows: *P < 0.05, **P < 0.01.

## Results

### Design of hemostatic microspheres with functional peptides

**Fig. 1:**
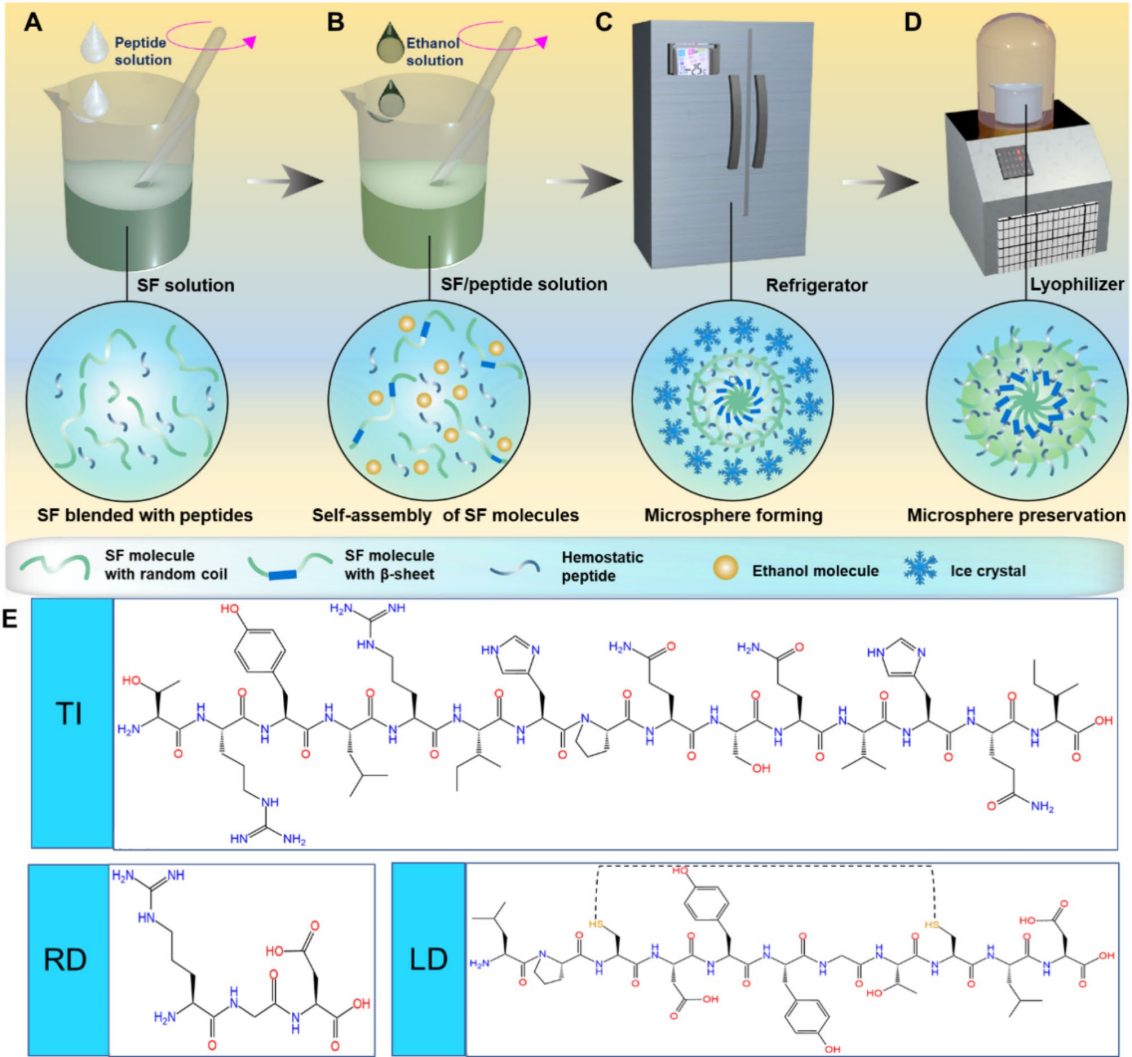
Preparation schematic of SF-based hemostatic microspheres. (**A**) Mixing fresh SF solution —initially in a random coil conformation- with the hemostatic peptide. (**B**) Ethanol is gradually added and mixed thoroughly to promote the nucleation and growth of β-sheets within the SF molecules. (**C**) The pre-nucleation system is then refrigerated at -20°C, where the formation of ice crystals impedes further growth of β- sheets, resulting in a microsphere capable of encapsulating hemostatic peptides. (**D**) The pre-frozen mixture undergoes freeze-drying to obtain platelet-like microspheres, which can be stored at 25°C for extended periods. (**E**) The molecular structure formulas of the three hemostatic peptides

To prepare microspheres that resemble platelets in size and morphology, we systematically investigated the effects of SF concentration, the ethanol/SF volume ratio, and self-assembly duration on the formation of SFMPs. Our results identified the optimal conditions for producing platelet-like SFMPs, characterized by an ethanol/SF volume ratio of 4:1 and a self-assembly time of 10 min (Fig. S1). Notably, during microsphere formation, we observed a transition in the secondary of SF from a random coil configuration to a more stable crystalline form (Fig. S2). To endow the microspheres with functionalities akin to those of platelets, we incorporated 3 distinct hemostatic peptides (RD, TI, or LD) with unique adhesion properties and hemostatic functions into the SFMPs (Fig. 1).

### Characterization of hemostatic microspheres

**Fig. 2:**
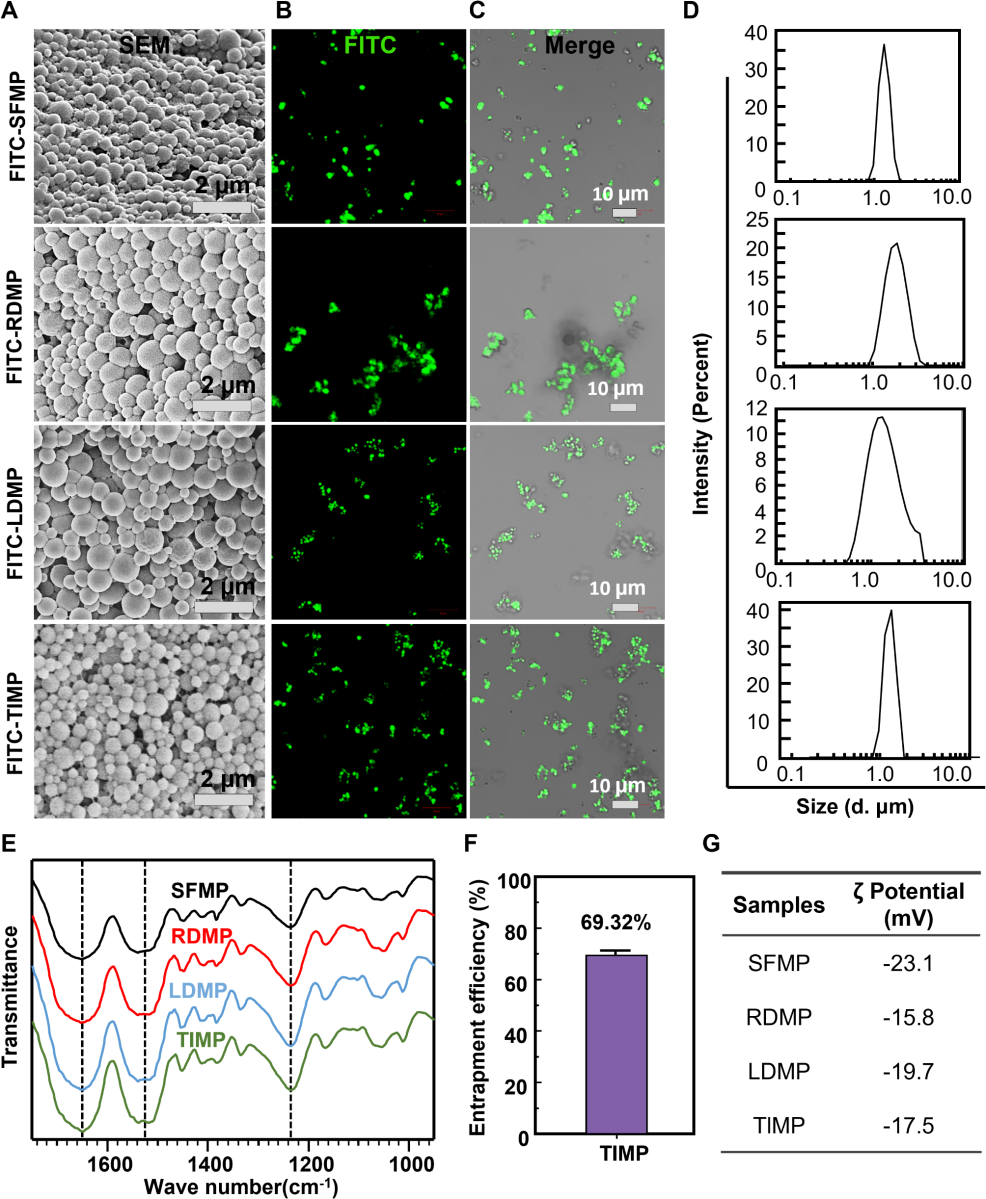
Characterization of hemostatic microspheres. (**A**) SEM of SFMP and hemostatic microspheres. **B**, **C**) CLSM observation of FITClabeled microspheres and hemostatic microspheres. (**B**) Green fluorescence shows the outline of the FITC-labelled microspheres; (**C**) Merged images combining bright-field and fluorescent channels. (**D**) Particle size distribution of the microspheres. (**E**) FTIR spectra of hemostatic microspheres. (**F**) Encapsulation efficiency of microsphere on the TI peptide. (**G**) Surface potential of hemostatic microspheres

The resulting hemostatic microspheres, as shown in Fig. 2A and D, and Fig. S3, had a diameter distribution between 1 and 3 μm, similar to the platelet, and could freely circulate in microvessels or capillaries in vivo without causing vessel blockage. To demonstrate the effective loading of peptides by SFMPs, FITC-grafted peptides were selected as a fluorescent probe to track peptide distribution within the microspheres (Fig. 2B, C). By comparing the fluorescence channel images (Fig. 2B) with merged bright field/fluorescence images (Fig. 2C), we observed that the FITC-carried hemostatic microspheres (RDMP, LDMP, TIMP) exhibited good monodispersity, with the fluorescence area almost completely overlapping with the microsphere outline. This observation, along with FTIR (Fig. 2E), confirms that SFMP can effectively bind and encapsulate low molecular weight peptides through a self-assembly mechanism, triggered by the transition of the SF molecular chains from an amorphous region to a crystalline region. The encapsulation efficiency of the microspheres for the TI peptide was calculated to be 69.32% (Fig. 2F), highlighting the microspheres’ ability to load hemostatic peptides. The surface potential distribution of SFMP, RDMP, LDMP, and TIMP was -23.1 mV, -15.8 mV, -19.7 mV, and -17.5 mV, respectively (Fig. 2G), indicating that all the microspheres maintained relatively stable electronegativity [41]. Therefore, the above results demonstrate that the MP can effectively load the hemostatic peptides and the hemostatic microspheres with morphology, size, and potential characteristics are similar to platelets.

### Cytocompatibility, cell adhesion and hemocompatibility

**Fig. 3:**
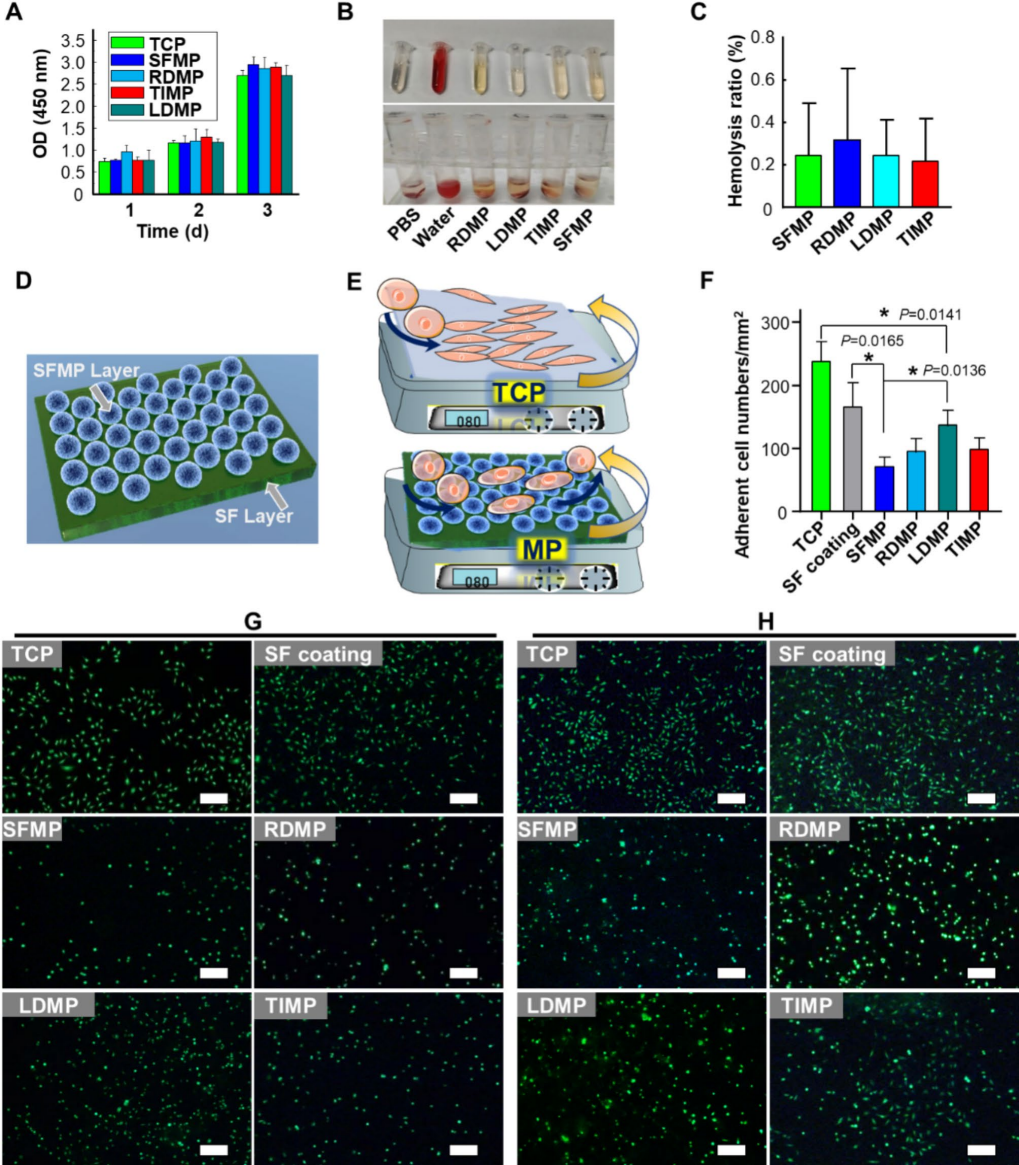
Assessment of cytocompatibility, hemocompatibility, and cell adhesion properties of hemostatic microspheres: (**A**) Cell viability of microspheres suspended in the culture medium assessed using CCK-8 assays (Mean ± SD, *n* = 3). **B**-**C**) Hemolysis evaluation of hemostatic microspheres; (**B**) visual representation of hemostatic microspheres in blood-containing tubes (top: supernatant after centrifugation; bottom: precipitates after centrifugation); (**C**) Hemolysis ratios of hemostatic microspheres (Mean ± SD, *n* = 3). (**D)** Schematic illustration of the bilayer microsphere film structure for evaluating cell adhesion and cytocompatibility, with the upper layer being a microsphere layer and the lower layer being a SF layer. (**E**) Diagram illustrating cells performing horizontal shaking on TCP and bilayer microspheres film to remove non-adherent cells. (**F**) Amount of cell adhesion to microsphere films at 12 h (Mean ± SD, *n* = 3). **G**-**H**) Micrographs showing cell adhesion on microsphere films; (**G**) 12 h; (**H**) 3 d; Scale bar: 200 μm

Biomaterials directly interact with the tissue environment, making their biocompatibility a crucial consideration. Here, we assessed the cytocompatibility of SFMPs using L929 cells and evaluated their hemocompatibility using rat blood. Figure 3A demonstrates that all microsphere groups exhibited similar cell growth activity to the control group after 1 and 2 d of cell culture, indicating good cytocompatibility based on the CCK8 assay. From day 2 to day 3, cell activity in each group increased rapidly, suggesting that the hemostatic microspheres promote cell growth. To visually observe the adhesion behavior between cells and microspheres, L929 cells were seeded onto a bilayer film structure coated with SF microspheres (Fig. 3D). After removing unadhered cells by shaking, live/dead cell staining was performed (Fig. 3E-H). The results showed bright green fluorescence (indicating live cells) in all groups, with almost no red fluorescence (indicating dead/apoptosis cells) after 12 h (Fig. 3G) and 3 d of cell culturing (Fig. 3H). These findings indicate that neither the SFMPs nor the hemostatic microspheres exhibit cytotoxicity. It is important to highlight that the number of adherent cells on all microsphere groups was significantly lower than that on tissue culture-treated plate (TCP) and SF coating groups at both 12 h (Fig. 3F, G) and 3 d (Fig. 3H) of cell seeding. This characteristic suggests that the microspheres have a low affinity for normal tissue cells, which could facilitate their free flow in blood vessels.

To evaluate the hemocompatibility of the microspheres in a blood environment, we conducted a hemostasis rate test (Fig. 3B-C). The SFMP group and all the hemostatic microsphere groups showed separation between the erythrocyte layer and the supernatant layer after centrifugation, indicating good hemocompatibility (Fig. 3B). In contrast, intact erythrocytes cannot be separated in the water-treated group due to hemolysis occurring. The HR of SFMP, RDMP, LDMP, and TIMP were 0.24%, 0.31%, 0.24%, and 0.22%, respectively (Fig. 3C). These rates are significantly lower than the maximum allowable hemolysis ratio specified by ISO 10993-4: 2017 (HR ≤ 5%), demonstrating that all microsphere solutions meet the safety requirements for clinical use. Therefore, the SFMP and hemostatic microspheres in this study exhibited good in vitro cytocompatibility and hemocompatibility.

### Platelet adhesion

**Fig. 4:**
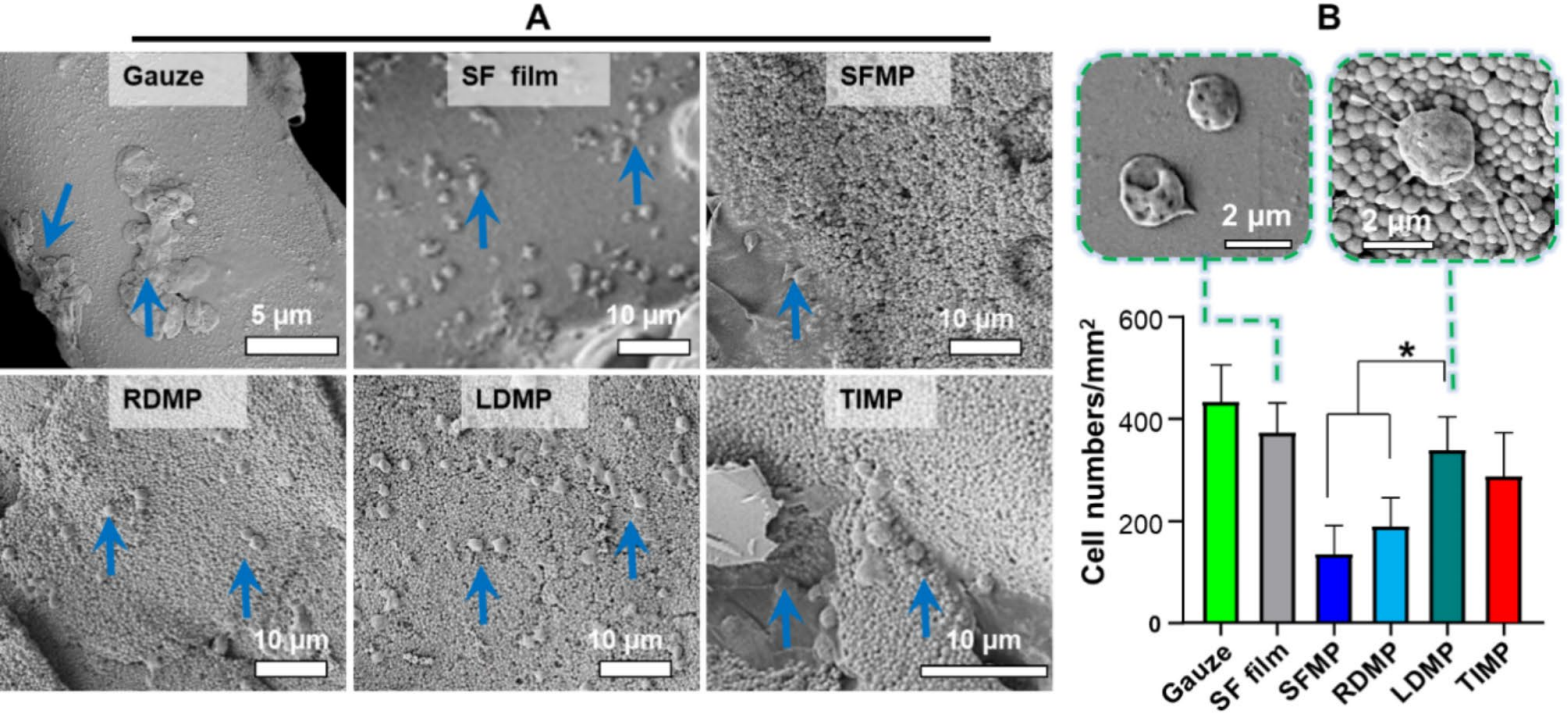
Representative SEM observation of platelet adhesion behavior on SFMP film and hemostatic microsphere films after 2 h. (**A**) Observation of platelets on microsphere films by SEM. (**B**) Quantitative analysis of platelets on microsphere films. Gauze and SF film were the control groups (Mean ± SD, *n* = 3)

When blood vessels or tissues are damaged in vivo, platelets immediately aggregate at the site of injury, initiating primary hemostasis. To investigate the effect of microspheres on platelet activation, we tested their impact on platelet adhesion and activation compared to gauze and a pure SF film control (Fig. 4). Figure 4A shows that the SFMP group had minimal platelet adhesion after 2 h, indicating low platelet binding ability. In contrast, the TIMP and LDMP groups exhibited strong adhesion ability to platelets after 2 h of incubation, with LDMP having the highest number of adhered platelets. Interestingly, we observed irregularly shaped platelets in the LDMP group (Fig. 4B), indicating that these platelets were activated. These results demonstrated that peptide-loaded microspheres enhance platelet adhesion, with LDMP being the most effective in promoting platelet adhesion and inducing activation.

### Fibrin polymerization and clot formation in vitro

**Fig. 5:**
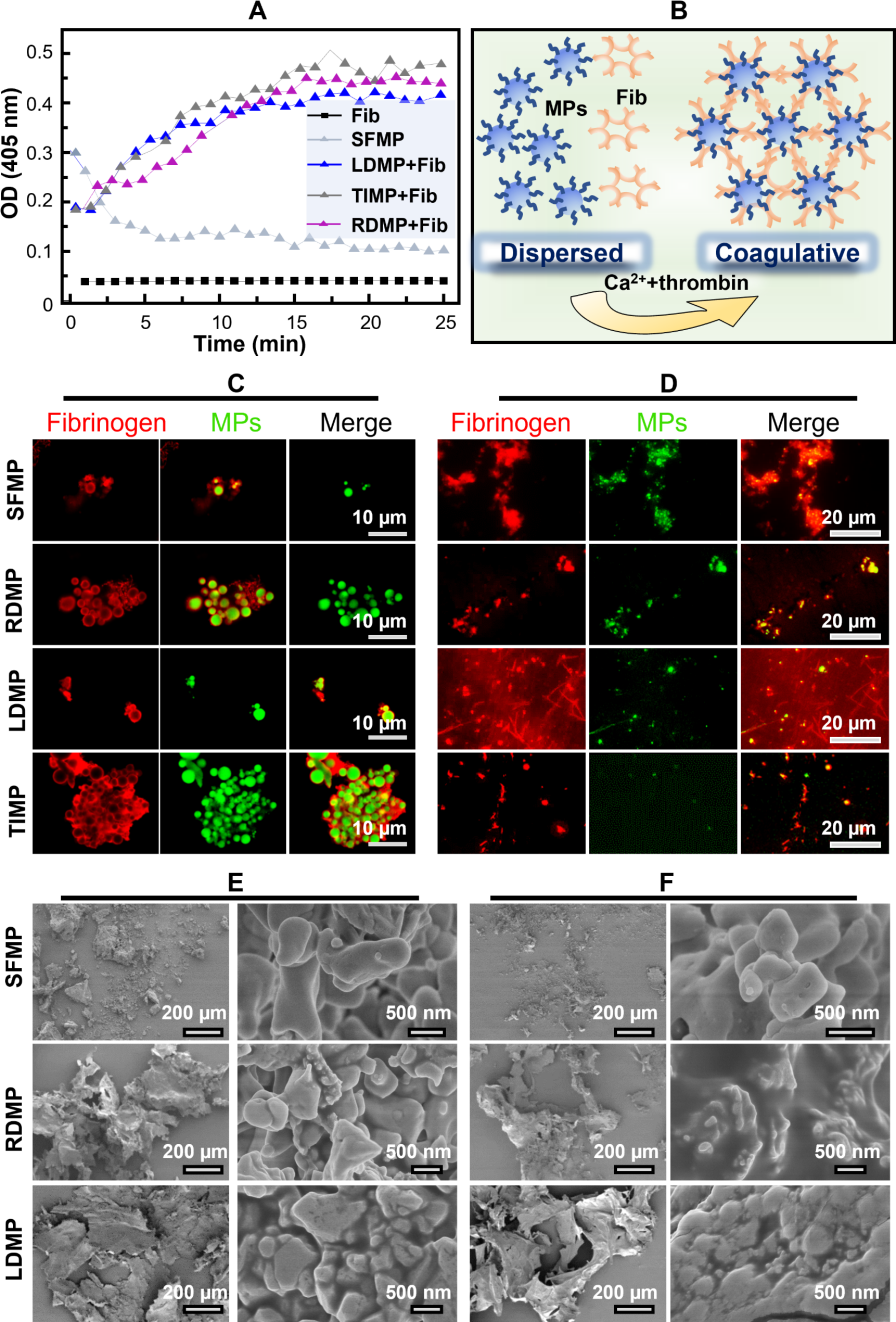
In vitro characterization of fibrin polymerization. (**A**) Binding kinetics of hemostatic microspheres and fibrin polymerization. (**B**) Schematic illustration of the self-assembly process of MPs and fibrinogen in simulated plasma. MPs: microspheres; Fib: fibrinogen. (**C**) Interaction between microspheres and fibrinogen without thrombin adding, red: fibrinogen; green: microspheres. (**D**) Activation reaction of microspheres after thrombin addition, red: fibrinogen; green: microspheres. E-F) SEM observation of hemostatic microspheres participating in fibrin polymerization at 25 °C (**E**) and 37 °C (**F**). SFMP induces fibrin formation at a relatively slow rate, while RDMP and LDMP promote the formation of intact membrane structures at 37 ℃. This is due to the promotion of fibrinogen activation by thrombin, leading to the binding of hemostatic microspheres and the formation of a cross-linked fibrin network

Secondary hemostasis is a complex coagulation process involving multiple coagulation factors that convert soluble fibrinogen in the blood into an insoluble fibrin network. To evaluate the involvement of microspheres in the thrombin activation process, a turbidity test was conducted (Fig. 5A, B). As shown in Fig. 5A, both the pure SFMP and the thrombin-only groups maintained a low optical density (OD) at the beginning of the reaction. In contrast, the OD value of the hemostatic microsphere groups increased gradually over time following the addition of fibrinogen and stabilized after 13 min, indicating their participation in the thrombin activation reaction. Besides, the fibrinogen activation reaction process was directly observed using CLSM (Fig. 5C, D). A layer of fibrinogen can be coated around the microspheres, but no fibrous or net structure was observed in any groups after 30 min of reaction. Interestingly, long filaments appeared in the LDMP group after the addition of thrombin, while shorter rod-shaped fibers appeared in the TIMP group (Fig. 5D). This is because LD is a fibrin-targeting peptide that rapidly enriches newly formed fibrin and facilitates its assembly into a fibrous structure (Fig. 5B). These results from the turbidity test and CLSM observations provide evidence that the hemostatic microspheres effectively promote fibrin polymerization.

Specific targeting of fibrin is a crucial characteristic of “smart” hemostatic agents, ensuring that they interact only with the wound site and not with precursor fibrinogen or unactivated platelets. The binding degree of hemostatic microspheres to fibrin at different temperatures was observed using SEM. As can be seen from the low magnification of SEM, sparse powder structures were observed in the SFMP group (Fig. 5E), indicating that the SFMP was not involved in the fibrinogen cross-linking process. In contrast, membrane-like structures were found in the RDMP and LDMP groups at 25°C. More importantly, the most intact membrane structure was observed in the LDMP group at 37℃ (Fig. 5F), indicating that LD peptides can promote fibrin polymerization by targeted interaction, leading to the binding of the LDMP to the fibrin network structure. Additionally, high-magnification SEM images revealed micro-particles of varying sizes and shapes (Fig. 5E, F), indicating that both SFMP and hemostatic microspheres adopt a flexible structure as platelets after being activated. These results demonstrate that hemostatic microspheres, especially LDMP, had a good ability to bind and promote fibrin polymerization.

**Fig. 6:**
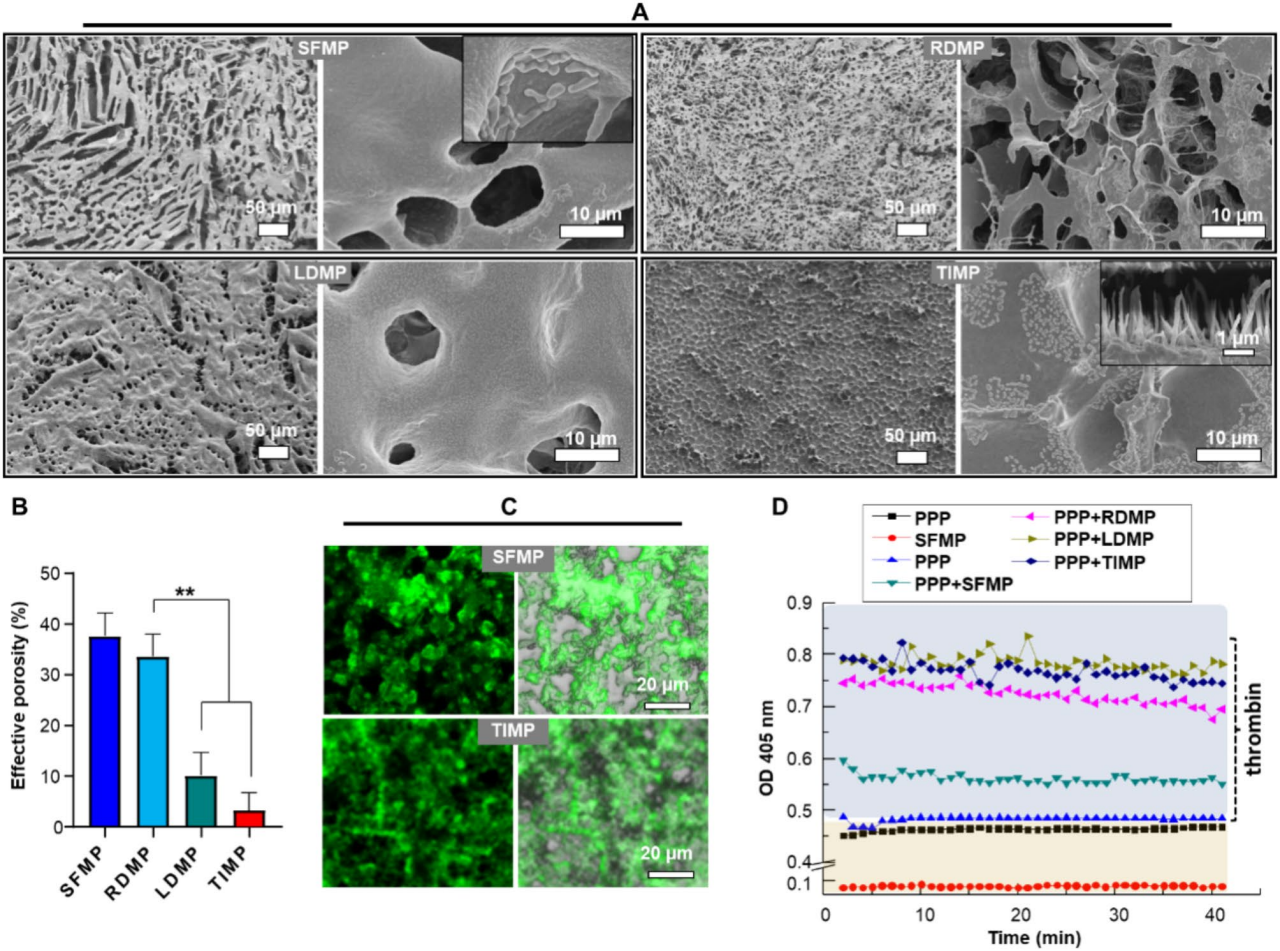
Hemostatic microspheres mediated blood clot formation in vitro. (**A**) SEM observation of microspheres after reaction with PPP. The upper right panel of the TIMP shows that the interior of the clot contains a large number of fibrous structures. (**B**) Porosity analysis of the membranes formed by the reaction of microspheres and PPP (Mean ± SD, *n* = 6). (**C**) CLSM of FITC labeled microspheres after the addition of PPP. (**D**) Kinetic test of the reaction of hemostatic microspheres with PPP

To further demonstrate the ability of hemostatic microspheres to participate in artificial clot formation in the absence of platelets, we conducted in vitro experiments using platelet-poor plasma (PPP). The reaction between microspheres and PPP was observed, and the results showed that aqueous SFMP/PPP exhibited a dispersed microsphere morphology (Fig. 6C), while dried SFMP/PPP had a loose and porous structure (Fig. 6A), indicating that SFMP maintained its independent spherical structure. Meanwhile, the dried RDMP/PPP and LDMP/PPP also had a porous structure on their surfaces, but with lower porosity and thicker walls compared to the SFMP/PPP group (Fig. 6A, B). Interestingly, the TIMP/PPP group showed a lack of porous structure (Fig. 6A, B) and instead had numerous filaments inside (Fig. 6A and C), indicating that TIMP can be attached to fibrin, and eventually form a thick blood clot structure. To assess the effect of microspheres on the coagulation rate of PPP, kinetic tests were performed in vitro (Fig. 6D). Viscous fibrin clots were immediately observed in the RDMP, LDMP, and TIMP groups, but not found in the SFMP and the control groups. This data confirms that the rapid clotting reaction can be attributed to the specific binding of hemostasis microspheres with clotting factors in plasma. Therefore, the above results prove that hemostatic microspheres, particularly LDMP and TIMP, exhibit excellent effects in promoting fibrin aggregation and blood coagulation in vitro.

### Vascular trauma targeting and degradation analysis in vitro

**Fig. 7:**
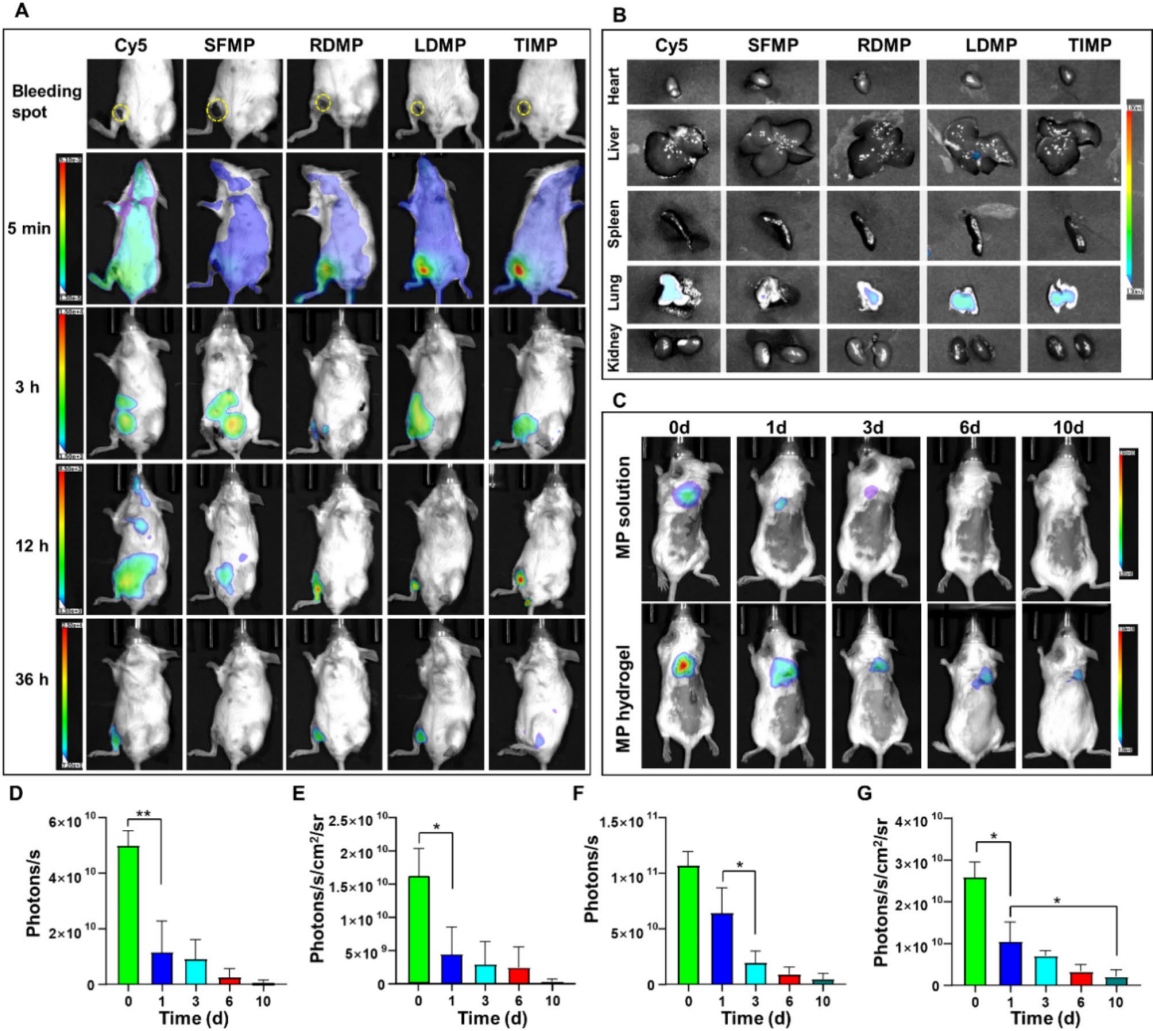
In vivo targeting and degradation studies of hemostatic microspheres. (**A**) Bioluminescence imaging to observe the targeting of microspheres at the wound site in vivo. (**B**) Ex vivo fluorescence imaging of the organs. (**C**) Biodegradation analysis of hemostatic microsphere aqueous solution (upper row) and hydrogel (lower row) in the dorsal side of mice. **D**-**E**) Total photon counts (**D**) and photon counts per unit time/area (**E**) in the region where the microsphere solution was injected (Mean ± SD, *n* = 3). **F**-**G**) Total photon counts (**F**) and photon counts per unit time/area (**G**) in the region where the microsphere hydrogel was injected (Mean ± SD, *n* = 3). Cy5: free Cy5; SFMP: Cy5-SFMP; RDMP: Cy5-RDMP; LDMP: Cy5-LDMP; TIMP: Cy5-TIMP

Subsequently, we investigated the in vivo targeting efficacy of hemostatic microspheres (Fig. 7A, B). Cy5-labeled SFMP and hemostatic microspheres were injected into the body to assess their ability to target the wound site. Compared to free Cy5 and Cy5-SFMP, the Cy5-RDMP, Cy5-LDMP, and Cy5-TIMP groups exhibited a strong fluorescence signal at the wound site within 5 min, with the Cy5-LDMP and Cy5-TIMP groups being the most pronounced signals (Fig. 7A). This phenomenon indicates that LDMP and TIMP have a faster and more effective targeting of the hemorrhagic site compared to SFMP. The fluorescence signal becomes more concentrated at the bleeding site after 3 h of observation, whereas the control group’s signal had not yet accumulated at the bleeding site (Fig. 7A). Also, microspheres not involved in hemostasis accumulated in metabolic organs, such as the kidneys, which are closer to the skin and thus produce a strong fluorescence signal in imaging. This may have resulted in the fluorescence signal from the metabolic organs being more prominent than that from the wound site. At 12 and 36 h post-injection, although the fluorescence signals in all groups diminished rapidly, small localized signals persisted at the bleeding site in the RDMP, LDMP, and TIMP groups. This prolonged retention implies enhanced binding affinity, allowing the hemostatic microspheres adequate time to exert their function, thereby effectively controlling bleeding. Furthermore, we observed the enrichment effects of microspheres in internal organs. Figure 7B shows that all microsphere groups had relatively low biodistribution in the heart, liver, and spleen. While, a small amount of enrichment was observed in the lung and kidney, possibly due to the dense vascular network in these regions.

Furthermore, to comprehensively analyze their in vivo degradation properties, microsphere solution was first injected subcutaneously and subsequently monitored (Fig. 7C-E). Immediately post-injection, a distinct fluorescence signal was observed, confirming the successful retention of the hemostatic microspheres beneath the skin. At 1 day post-injection, although signals from microspheres were detectable, the fluorescence intensity of the SFMP solution group declined by approximately 71% (Fig. 7D-E). To simulate the degradation of microspheres at the site of blood clots in vivo, the hemostatic microsphere hydrogel was injected similarly. The degradation characteristics were similar to those of the microsphere solution, with the hydrogel gradually degrading over time. Specifically, the fluorescence signal of the SFMP hydrogel group decreased by about 48% after 2 h injection (Fig. S4) and by approximately 60% at 1 day post-injection (Fig. 7F-G). These results indicate that, compared to the SF hydrogel group, the SFMP solution degrades more rapidly in vivo due to their increased fluidity of the solutions. Six days after injection, the fluorescent signals at the injection site were nearly absent, indicating near-complete degradation of the microspheres in vivo (Fig. 7D-G).

### Metabolic distribution and biocompatibility of hemostatic microspheres in vivo

**Fig. 8:**
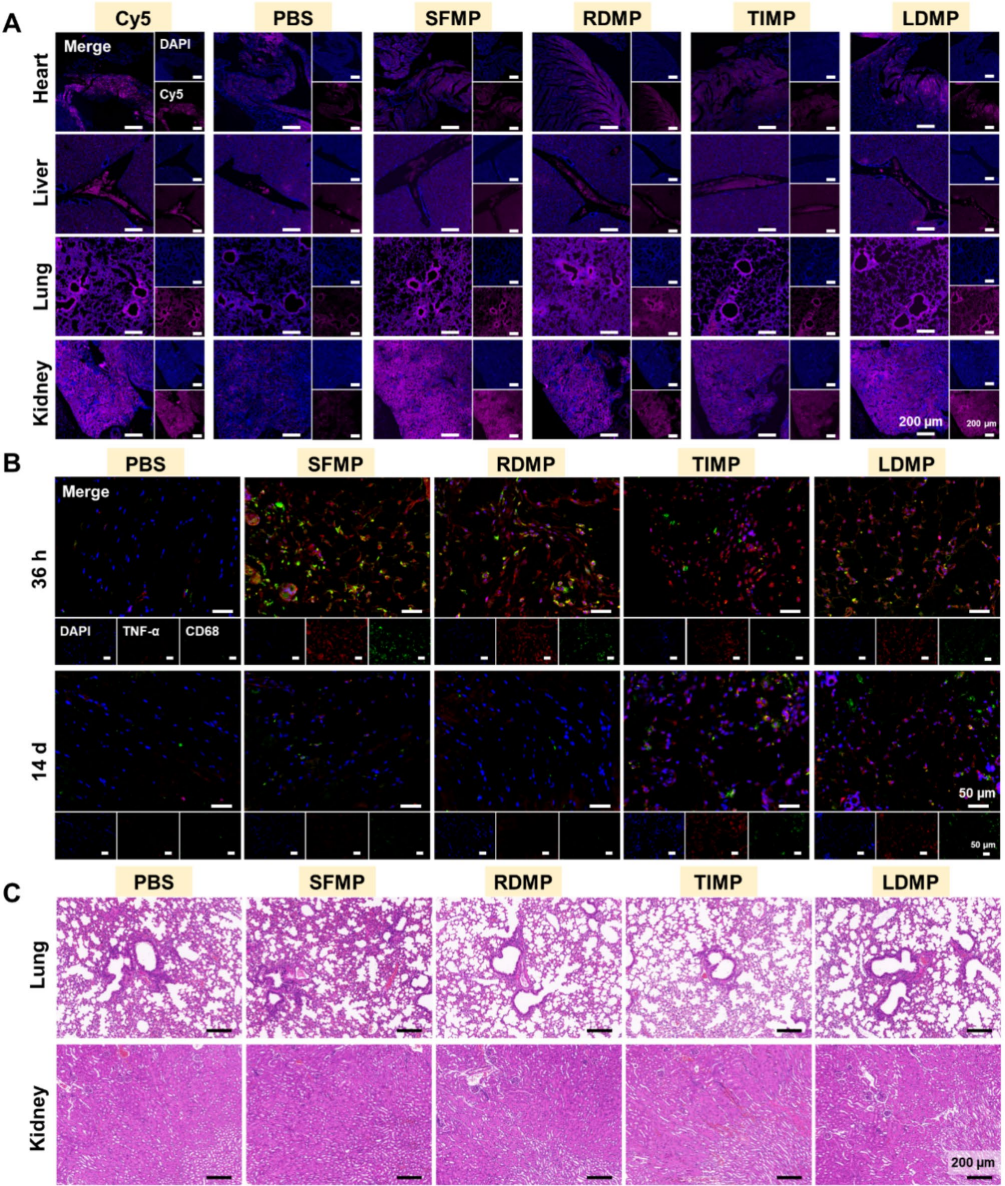
Metabolic distribution and biocompatibility of hemostatic microspheres in vivo. (**A**) Bioluminescence imaging illustrates the metabolic distribution of free Cy5 and Cy5-labeled microspheres in various metabolic organs 36 h post-injection. Cy5: free Cy5; SFMP: Cy5-SFMP; RDMP: Cy5-RDMP; LDMP: Cy5-LDMP; TIMP: Cy5-TIMP. (**B**) Immunofluorescence co-staining of tissue at the injection site, demonstrating the presence of inflammatory cells, highlighted by CD68 (green), and the pro-inflammatory cytokine TNF-α (red), with DAPI (blue) marking the cell nuclei. (**C**) H&E staining was performed on the collected tissues 14 d after injection of the hemostatic microspheres

Fig. 8A indicated that free Cy5 primarily accumulates in the liver, lungs and kidneys 36 h post-injection. While the distribution of the microspheres in the heart and liver appeared minimal, notable accumulation in the kidneys and lungs indicates that the microspheres may undergo metabolic processing predominantly in these tissues. In addition, the biocompatibility of hemostatic microspheres was further assessed through histological examination using H&E staining (Fig. S5, Fig. 8C). The results demonstrate that hemostatic microspheres have no detrimental effects on the structural integrity of heart, liver, lung, and kidney in the short (36 h) and the long term (14d), as shown by the well-preserved organ morphology and absence of pathological changes in each group.

To further investigate potential inflammatory responses elicited by the microspheres, we employed immunofluorescence co-staining (Fig. 8B) for the macrophage marker CD68 (green) and the pro-inflammatory cytokine TNF-α (red). The results demonstrated a presence of CD68 and TNF-α-positive cells after 36 h post-injection, indicating cellular infiltration consistent with an inflammatory response. This suggests that the degradation of the microspheres may actively involve inflammatory cells, which typically contribute to phagocytosis and subsequent tissue remodeling processes. In conclusion, these results demonstrate that these hemostatic microspheres can induce a slight localized inflammatory response in the short term, and possess good biocompatibility in the long term. The findings from this in vivo study provide crucial insights into the metabolic distribution, tissue compatibility, and potential inflammatory responses associated with the microspheres, establishing a foundation for their future application in promoting hemostasis and enhancing wound healing.

### Hemostatic effect of hemostatic microspheres in vivo

To assess the hemostatic effect of the microspheres in vivo, we constructed a mouse tail amputation model (Fig. S6). The results showed that LDMP and TIMP completely inhibit tail bleeding within 100 s, while the SFMP failed to achieve complete hemostasis even after 5 min. This indicates that LDMP and TIMP are highly effective in promoting rapid coagulation in traumatic injuries in vivo.

**Fig. 9:**
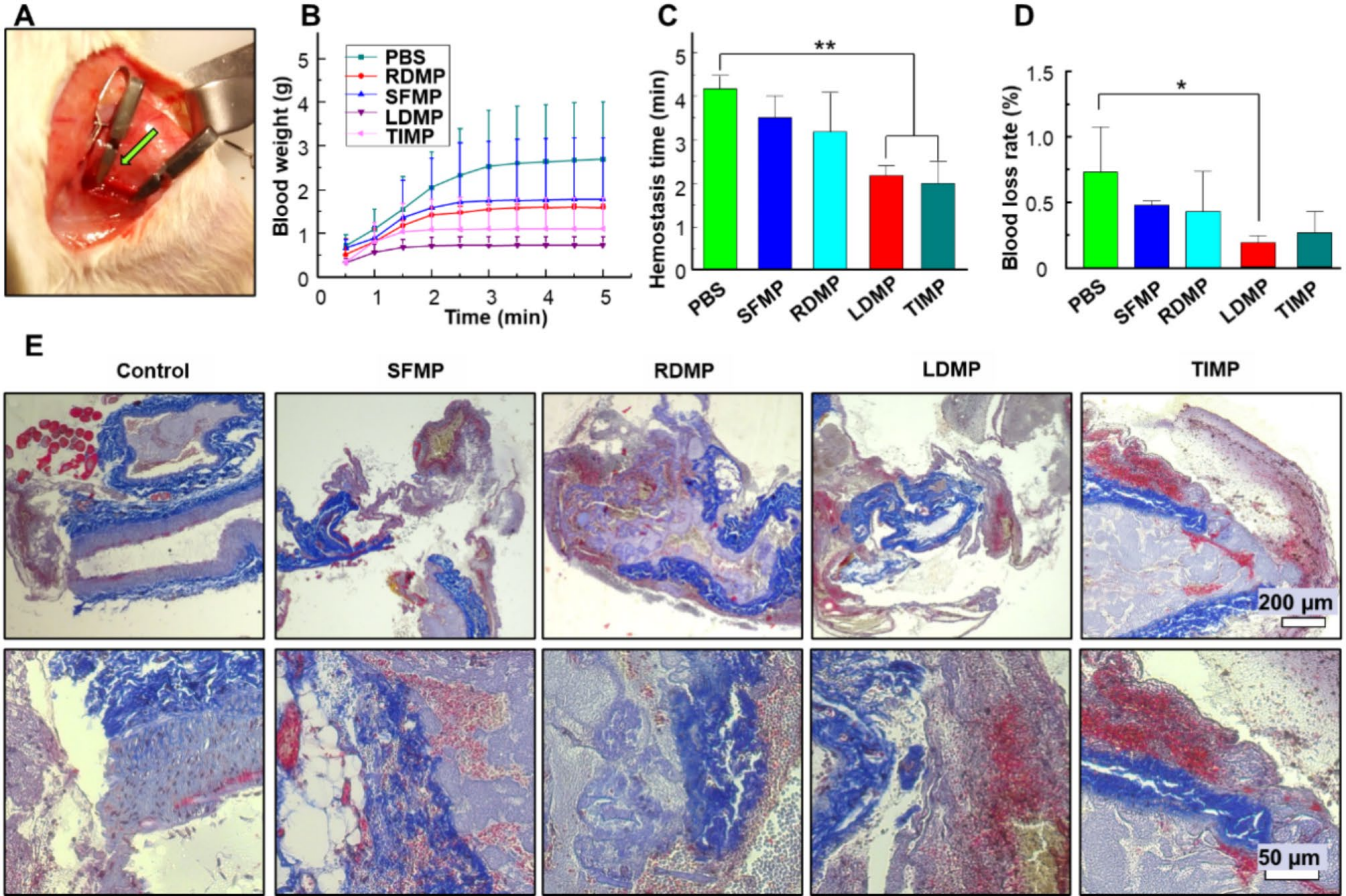
Hemostatic microspheres promote blood coagulation in vivo. (**A**) Digital photograph showing the construction of a rat femoral vein defect model. (**B**) Accumulated blood loss over time (Mean ± SD, *n* = 3). (**C**) Recorded hemostasis time during the hemostatic process (Mean ± SD, *n* = 3). (**D**) Blood loss rate (Mean ± SD, *n* = 3). (**E**) MSB staining of the injured veins (upper row: low-resolution images; bottom row: high-resolution images). Blue staining indicates the collagen or elastic fibers in the blood tissues. Blue-gray staining is the nucleus, and yellow staining shows the erythrocytes. Red represents the newly formed fibrin after bleeding. Both the TIMPs and LDMPs show significant red staining areas compared to the control and the SFMP

Furthermore, certain conditions such as inflammation, bacterial infection, pregnancy-induced hypertension, and hemophilia, can lead to deep tissue bleeding. Currently, there are limited non-invasive hemostatic materials available for treating deep tissue bleeding. To address this gap, we evaluated the targeting and coagulation effects of hemostatic microspheres using a rat femoral vein injury model (Fig. 9A). The SFMPs were injected prior to the injury to allow for circulation. We observed that the PBS group had the longest coagulation time of 4.17 min and the highest bleeding amount of 2.7 g (Fig. 9B, C). In contrast, the clotting time was shorter in the SFMP and RDMP groups (3.5 min and 3.4 min, respectively) compared to the PBS group. Surprisingly, the TIMP and LDMP groups achieved even shorter hemostatic times of approximately 2.0 and 2.4 min, respectively. Importantly, the LDMP group reduced bleeding volume by 74% compared to the SFMP group. Similarly, Fig. 9D revealed that the TIMP and LDMP groups had a lower blood loss rate (0.26wt%) compared to the PBS group, which had the highest rate at 0.78wt%. These results clearly indicate that LDMP and TIMP exhibit superior hemostatic performance in vivo.

Additionally, MSB staining was conducted to detect newly formed fibrin clots at the injured tissues (Fig. 9E). Large amounts of newly formed fibrin were observed around the damaged vessel site in the TIMP and LDMP groups, proving that TIMP and LDMP can accumulate at the trauma site and promote massive blood clot production. The hemostatic microspheres exhibited adherence to the surface of injured vascular endothelial tissues and enhanced clot formation at the injured site (Fig. 9E), thus demonstrating the potential of our peptide-based SFMP as a non-invasive coagulant in vivo. Therefore, the flexibility and platelet-like properties of these microspheres, enable their circulation in blood vessels and accumulation at sites of activated platelets and fibrin formation. This dynamic behavior facilitates the rapid formation of clots and efficient hemostasis in cases of significant vascular injury.

## Discussion

The development of hemostatic agents, particularly platelet-like microspheres, has garnered significant interest due to their promising hemostatic properties [17]. In this study, we aimed to evaluate the efficacy of SFMPs loaded with biologically active peptides to enhance the hemostatic process. The unique biocompatibility and biodegradability of SF render these hemostatic microspheres an attractive alternative to traditional platelet-like hemostatic agents. Our findings highlight the roles of these hemostatic microspheres in promoting coagulation (Fig. 9) while also raising important considerations regarding their performance in vivo (Figs. 7 and 8). Compared to newly developed [42] or commercial hemostatic agents [43] that achieve hemostasis in approximately 2–5 min, our results indicate that TIMP and LDMP exhibit remarkable coagulation-promoting effects during blood circulation.

To investigate the impact of hemostatic microspheres on the coagulation process in vitro, platelet-poor plasma (PPP) was isolated from whole blood (Fig. 6). This methodological approach allows us to isolate the specific effects of fibrinogen and other coagulation factors without the confounding influence of platelets. By removing platelets, we could delineate the contributions of our hemostatic microspheres in modulating the coagulation cascade. This work has clinical relevance for patients with impaired platelet function, such as those undergoing antiplatelet therapy or suffering from thrombocytopenia [44]. Understanding how hemostatic microspheres-based therapies work in a platelet-poor environment can help inform therapeutic strategies for enhancing hemostatic efficacy in these clinical scenarios.

The SEM images show that SFMPs do not significantly participate in fibrinogen crosslinking (Fig. 5E, F). One structural feature of SFMPs is their relatively smooth and non-adhesive surface (Fig. 2A), which contrasts with peptide-based hemostatic microspheres that possess specific receptors and polar amino acids that facilitate strong interactions with fibrinogen. The lack of such features in SFMP can hinder effective platelet binding (Fig. 4) and fibrinogen crosslinking reactions (Fig. 5E, F), and even impede cell adhesion (Fig. 3E-F). These limited interactions can elucidate the necessity for surface modifications (such as hemostatic peptides) to enhance their functionality as effective platelet mimetics. While SFMPs may not actively engage in fibrin crosslinking, their biomimetic properties as platelet substitutes could still be advantageous in certain contexts, as they interact with other components in the coagulation cascade.

The targeting efficacy of RGD-hemostatic microspheres is closely related to their coagulation function in vivo. However, RGD interacts with a range of substances beyond the hemostatic system [45]. Our research aims to elucidate the impact of broad versus specific binding on the hemostatic through the use of microspheres loaded with different peptides. Our findings indicate that RDMPs exhibit a degree of coagulation activity in vitro (Fig. 5); however, their hemostatic efficacy diminishes in vivo (Fig. 9). The integrin-binding capability of RGD peptides allows effective targeting of multiple cell types, such as endothelial cells, which may attenuate the hemostatic effect due to their non-specific targeting nature [45]. Furthermore, RGD peptides can inhibit platelet aggregation by binding to platelet glycoprotein (GP) IIb/IIIa receptors [37]. Consequently, the design and evaluation of the RDMP group are crucial for evaluating the coagulation efficacy of MPs.

The hemostatic microspheres also demonstrate significant promise regarding long-term biocompatibility in vivo (Fig. 8B, C). Their simple preparation method and the use of mild, natural materials contribute to a safer profile, minimizing the risk of adverse reactions commonly seen with synthetic alternatives. Preliminary results indicate good biocompatibility, suggesting that the microspheres can effectively integrate with host tissues without eliciting significant inflammatory responses (Fig. 8). Our research demonstrates that SFMP hydrogel lost 48% under the skin within 2 h (Fig. S4), proving their rapid metabolic ability in non-clotting sites. Similar results were obtained by Gao et al., who conducted a pharmacokinetic analysis of hyaluronic acid–vWF–binding peptide conjugates, finding that they degraded to half their initial concentration in 0.9 h [46]. Their ability to biodegrade in vivo is advantageous for long-term applications, as it reduces the potential for chronic foreign body reactions that are commonly seen with many synthetic hemostatic agents. Notably, the degradation products of the hemostatic microspheres are beneficial small peptides or amino acids for the organism [26]. In contrast to other hemostatic products, which may rely on complex manufacturing processes and potentially reactive materials, these favorable degradation characteristics of the SF-based microspheres may enhance their acceptance within the body, leading to improved healing outcomes over time.

Despite the promising characteristics of these hemostatic microspheres, several limitations must be addressed to advance these hemostatic agents. One significant concern is the potential for variable hemostatic efficacy due to the potential impact of partial peptide release, which may impact the microspheres’ performance in clinical settings. Additionally, a thorough evaluation of wound characteristics (size, depth, severity, and tissue type) is essential for assessing the actual performance of these hemostatic microspheres. Variabilities in wound characteristics could result in differing hemostatic outcomes and necessitate rigorous testing across diverse scenarios to establish reliable efficacy. Additionally, concerns regarding long-term biosafety and biodegradation in vivo remain crucial.

## Conclusion

The design and preparation of hemostatic materials that mimic the morphology and physiological function of platelets are essential for first-aid and clinical applications[. In this study, we successfully prepared uniform platelet-like SF-based microspheres using a one-pot method. Furthermore, we incorporated three hemostatic peptides with distinct tissue targeting functions into SFMPs to comprehensively evaluate their hemostatic properties. Our results demonstrated that the SF molecular chain effectively locks the hemostatic peptides during the freezing self-assembly, remaining stable throughout the microsphere formation. The resulting hemostatic microspheres exhibited remarkable abilities to recruit platelets, enhanced fibrin binding, and accelerated fibrin clot formation, thereby displaying excellent hemostatic effects. Notably, our findings revealed that the hemostatic microspheres effectively promote rapid targeting of bleeding sites, facilitate hemostasis, and significantly reduce bleeding volumes in vivo. Specifically, LDMP targets fibrinogen, facilitating its aggregation and thereby promoting hemostasis. Meanwhile, TIMP binds to activated platelets and polymerized fibrinogen, triggering hemostatic responses. These microspheres hold great potential for achieving rapid clotting at superficial traumatic bleeding sites and are also expected to be utilized for targeted hemostasis in cases of deep bleeding. Moreover, the production process of these microspheres does not involve the use of toxic chemical reagents and utilizes natural proteins and peptides, making them safe and biocompatible with broad clinical application prospects in biomedicine.

## Electronic supplementary material

Below is the link to the electronic supplementary material.

Supplementary Material 1

## Data Availability

No datasets were generated or analysed during the current study.
