# Supplementary material for: Injectable platelet-mimicking silk protein-peptide conjugate microspheres for hemostasis modulation and targeted treatment of internal bleeding
Source: J Nanobiotechnology. 2025 Feb 20;23:128. doi: 10.1186/s12951-025-03180-w (PMC11844073; doi:10.1186/s12951-025-03180-w)
Supplement: Supplementary file 1 — Supplementary Material 1 [file 12951_2025_3180_MOESM1_ESM.docx]

**Injectable Platelet-Mimicking Silk Protein-Peptide Conjugate Microspheres for Hemostasis Modulation** **and Targeted Treatment of Internal Bleeding**

Yajun Shuai^1,2^, Yu Qian^1^, Meidan Zheng^1^, Chi Yan^1^, Jue Wang^1^, Peng Wang^1^, Jie Wang^1,2^, Chuanbin Mao^3^*, Mingying Yang^1,2^*

^1^Institute of Applied Bioresource Research

College of Animal Science

Zhejiang University

Hangzhou 310058, China

^2^Key Laboratory of Silkworm and Bee Resource Utilization and Innovation of Zhejiang Province

Zhejiang University

Hangzhou 310058, China

^3^Department of Biomedical Engineering

The Chinese University of Hong Kong

Sha Tin, Hong Kong SAR, China

*E-mail: cmao@cuhk.edu.hk

*E-mail: yangm@zju.edu.cn

**Supplementary** **data**


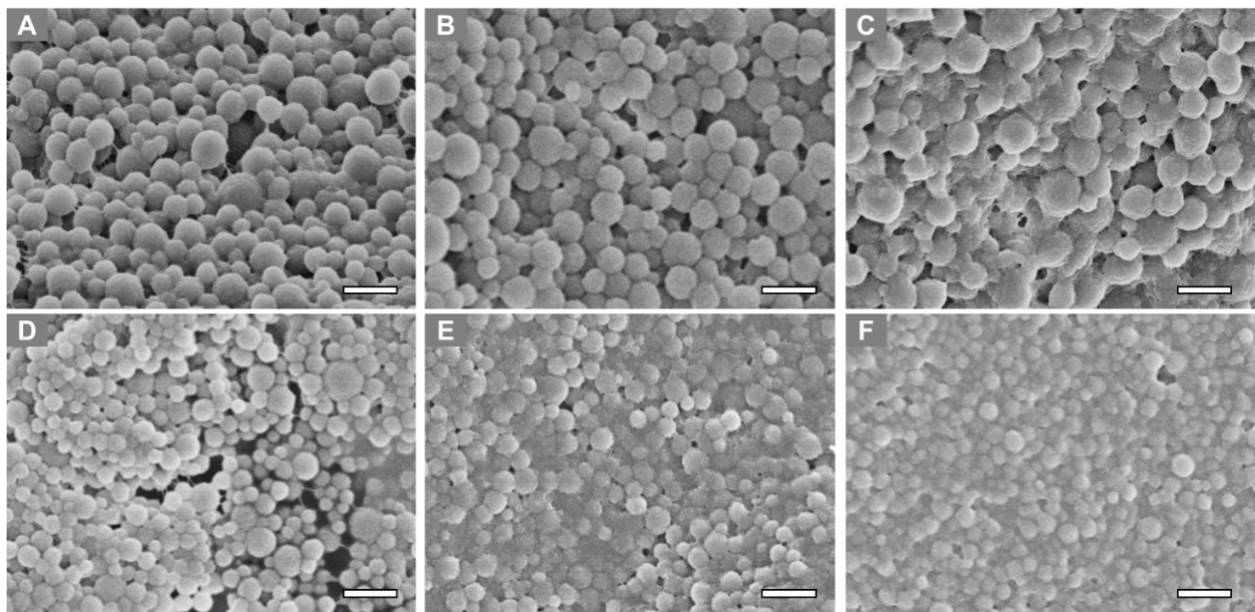


**Fig. S1.** SEM images of SFMP were prepared under different conditions. A-C: 1 mL (A), 2 mL (B), or 4 mL (C) of ethanol was added to 8 mL of 2% SF solution, respectively; D-F: The self-assembly time was set as 10 min (D), 30 min (E), and 50 min (F), respectively. Scale bar: 1 μm.


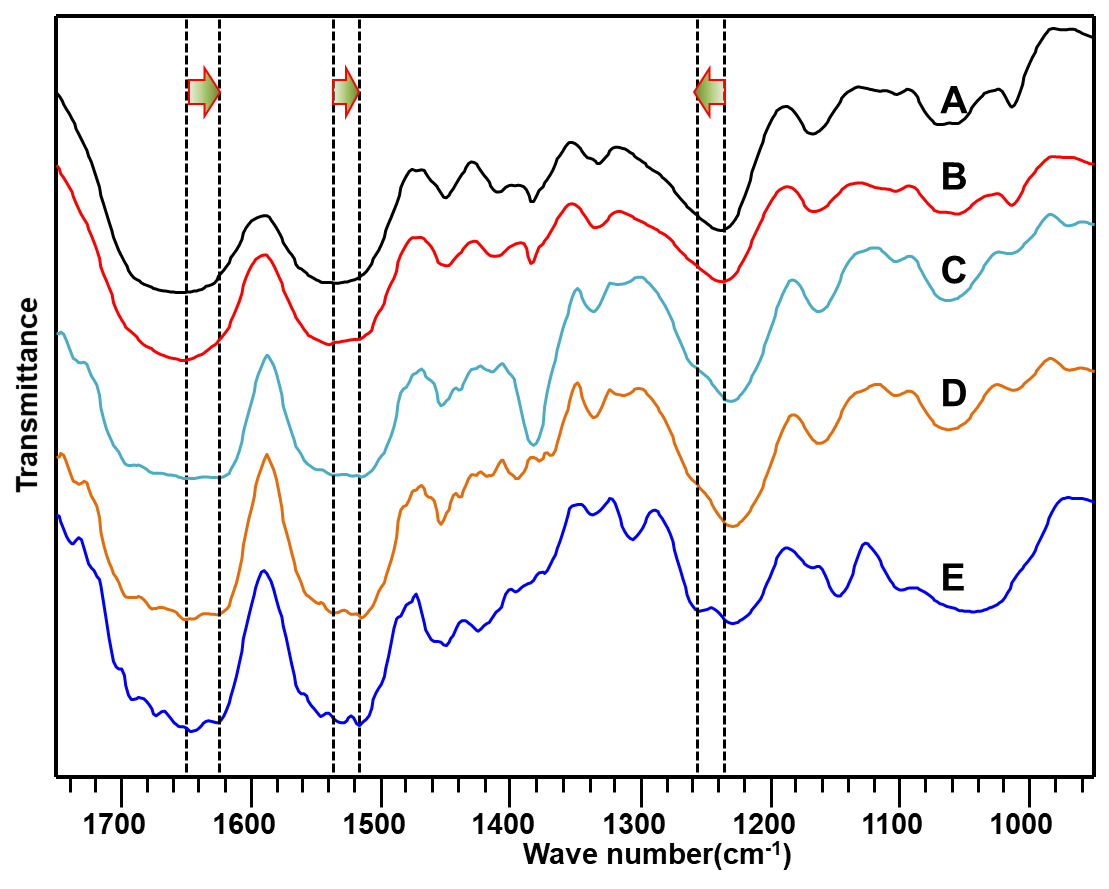


**Fig. S2**. FTIR spectra of SFMP powders. (A) dry SF powder, (B-D) SFMP powders prepared from different ethanol/SF ratios and self-assembly time. (B, D) The ethanol/SF ratio was 1:4; (C, E) ethanol/SF ratio was 1:2. The self-assembly time was set at 10 min (B), 30 min (C), and 50 min (D, E).


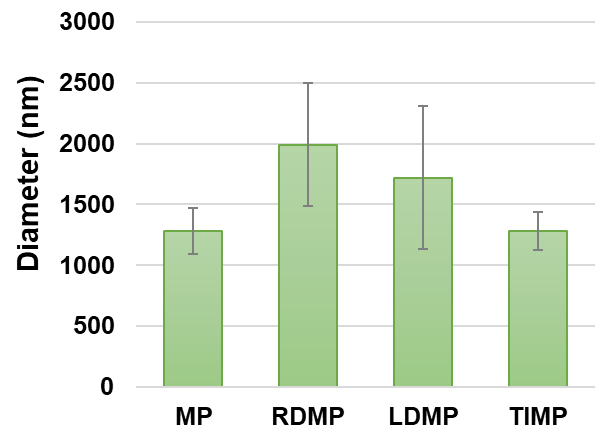


**Fig. S3**. Statistics of the diameter of different microspheres.


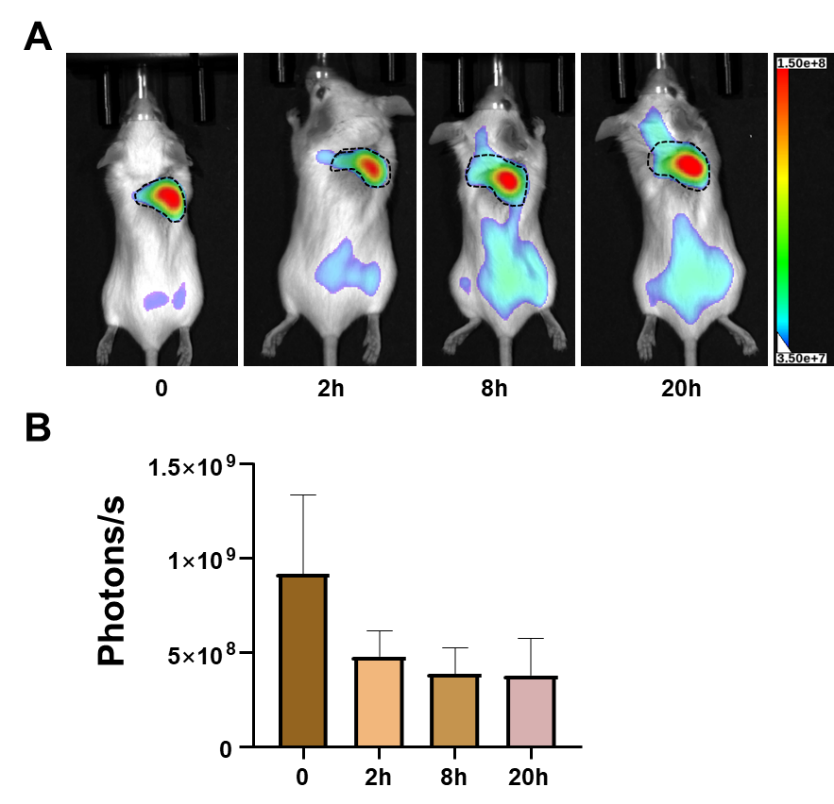


**Fig. S4**. (A) Biodegradation analysis of hemostatic microsphere hydrogel in the ROI areas of mice within 20 h. (B) Total photon counts in the ROI areas (Mean ± SD, n=3).


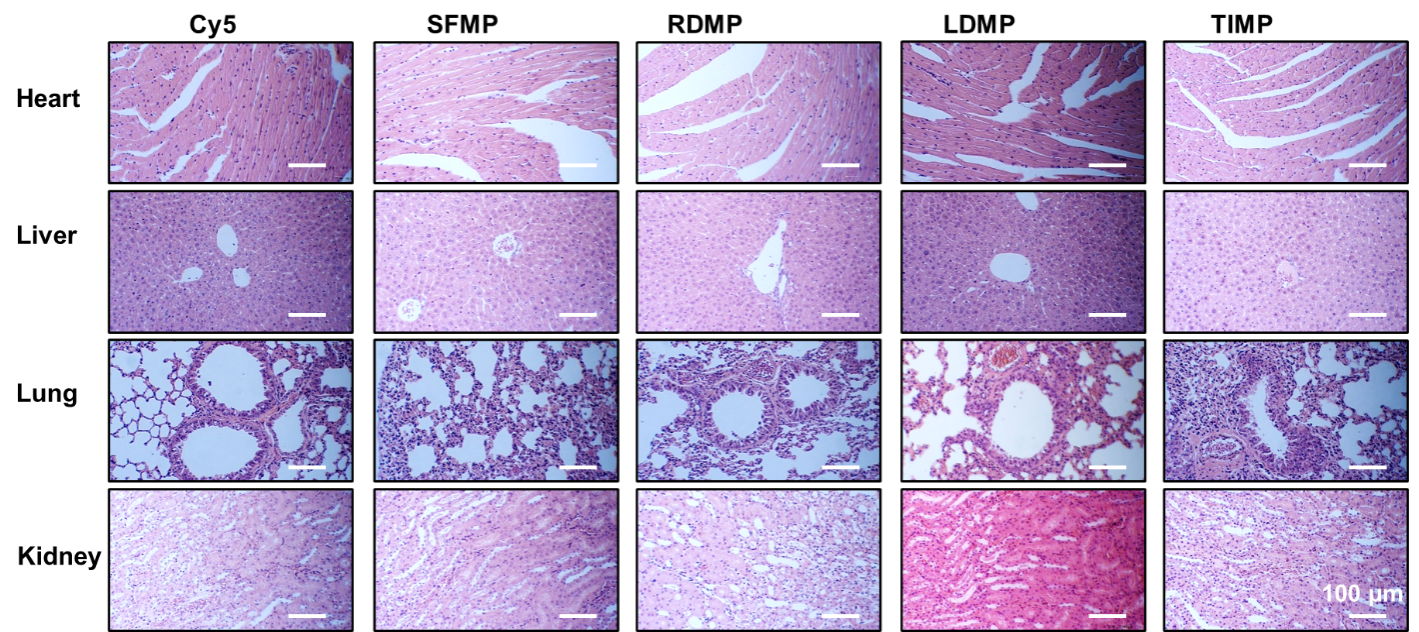


**Fig. S5.** H&E staining was performed at 36 h after injection of the hemostatic microspheres.

**
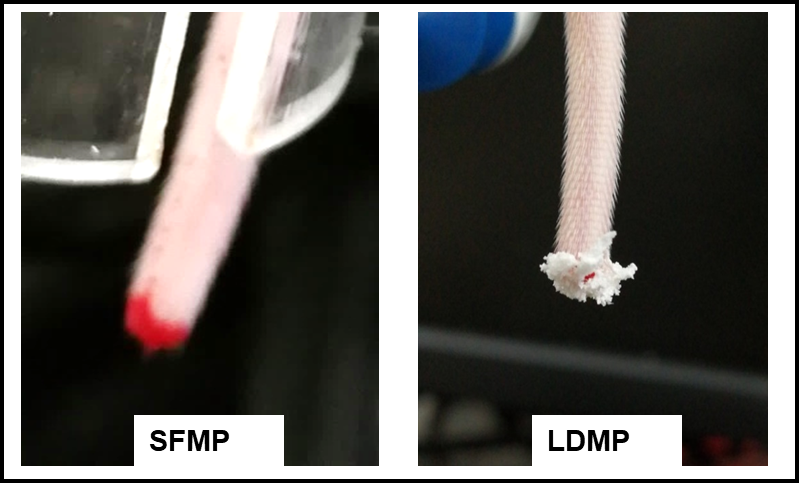
**

**Fig. S6.** A mouse tail amputation model was used to verify the hemostatic effect of hemostatic microspheres. After 100 s, the mouse tails in the SFMP group were still bleeding, while the mouse tails in the LDMP group had completely stopped bleeding.
